# Meteorological Table

**Published:** 1813-05

**Authors:** 


### METEOROLOGICAL TABLE.

### *From March 25, to April 25, 1813.*

| | D. | Therm. | | | Barom. | | Hygrom. Dry.— | | | —Damp. | | | Winds. | Atmos. Variation. |
|---|---|---|---|---|---|---|---|---|---|---|---|---|---|---|
| | 26 | 47 | 51 | 44 | $30^4$ | 5 | 5 | — | 7 | | | | W | F.. |
| | 27 | 48 | 55 | 50 | — | — | 10 | 12 | 6 | | | | SW | F... |
| | 28 | 47 | 69 | 58 | $30^4$ | — | 10 | 5 | 1 | | | | NW | F.. |
| | 29 | 56 | 60 | 55 | $30^2$ | — | 1 | — | — | | | | W | F.. |
| | 30 | 53 | 57 | 50 | $30^1$ | — | | | | | | | W | F. R. F. |
| | 31 | 50 | 55 | 46 | $29^8$ | 6 | 4 | 7 | — | | | | W | C.. F.. C. |
| ● | 1 | 40 | 38 | 41 | $29^8$ | — | 1 | — | — | | | | SW | R.. |
| | 2 | 42 | 45 | 40 | $29^4$ | — | 1 | 3 | 4 | | | | W | C.. Hail.. R... C.. |
| | 3 | 37 | 39 | 38 | $29^6$ | 7 | 6 | 7 | — | | | | W | F. Hail... C.. |
| | 4 | 40 | 44 | 41 | $29^8$ | 9 | 8 | 9 | — | | | | NW | F. |
| | 5 | 41 | 43 | 42 | $29^8$ | — | 6 | 2 | — | — | — | 1 | W | C.. R.. |
| | 6 | 50 | 55 | 52 | $29^9$ | — | | | | 1 | 2 | 4 | W | C.. |
| ◐ | 7 | 50 | 54 | 51 | 30 | — | | | | 2 | 3 | 2 | SW | C. |
| | 8 | 56 | 59 | 55 | — | — | 4 | 5 | — | | | | SW | F.... |
| | 9 | 55 | 67 | 56 | — | — | 10 | — | — | | | | W | F.... |
| | 10 | 56 | 68 | 54 | $30^1$ | — | 13 | 15 | 19 | | | | W | F.... |
| | 11 | 55 | 67 | 51 | — | — | 15 | — | 18 | | | | SW | F.... |
| | 12 | 48 | 66 | 56 | $30^2$ | — | 15 | 14 | — | | | | W | F.... |
| | 13 | 53 | 66 | 52 | $30^3$ | 4 | 15 | — | 19 | | | | W | F.. |
| | 14 | 49 | 64 | 50 | $30^3$ | — | 20 | — | — | | | | SW | C.. F... |
| ○ | 15 | 50 | 69 | 52 | $30^1$ | 30 | 30 | 37 | 20 | | | | SW | C. F.... |
| | 16 | 55 | 61 | 56 | — | — | 28 | 30 | 17 | | | | W | F. |
| | 17 | 54 | 64 | 51 | $29^9$ | — | 15 | 20 | 27 | | | | NW | R.. F.. |
| | 18 | 55 | 60 | 53 | 30 | $30^1$ | 19 | 30 | 36 | | | | W | F... |
| | 19 | 56 | 64 | 57 | $30^2$ | — | 15 | 19 | 21 | | | | N | F... |
| | 20 | 58 | 66 | 54 | $30^1$ | — | 23 | 40 | 35 | | | | W | F.... |
| | 21 | 48 | 58 | 46 | — | — | 15 | 19 | 32 | | | | NE | C.. |
| | 22 | 44 | 47 | 42 | — | — | 17 | 20 | 25 | | | | NE | C.. Snow. R.. C.. |
| ◑ | 23 | 45 | 49 | 42 | $30^2$ | — | 20 | — | 23 | | | | N | C.. Hail.. R.. C... |
| | 24 | 44 | 45 | 42 | 30 | — | 20 | — | 25 | | | | NE... | C.. |
| | 25 | 45 | 50 | 46 | $29^9$ | 8 | 23 | 17 | 15 | | | | SE | C.. R.. C.. |

Quantity of rain from the 25th of March to the 25th of *April*, $\frac{53}{100}$ of an inch.

The characteristic of this interval, like that of the preceding, has been an unusual calmness, with a remarkably dry atmosphere: the quantity of rain is precisely the same with that of the interval from the 25th of February to the 25th of March. During the greater portion of the present period, comprehending a part of March and nearly the whole of April, the temperature has been high, the thermometer sometimes rising to 69, with a pleasant softness of the air; but from the 20th of April a great change has occurred. A small quantity of snow has fallen, and frequent showers of hail, with a quality of cold in the atmosphere, producing an effect on the feelings much greater than the depression of the thermometer, *à priori*, would indicate.

Catarrh has prevailed very extensively, and some cases of scarlatina anginosa, and synanche tonsillaris, have occurred; ophthalmia also appeared in some districts.

NOTICES

